# Pituitary Stalk Interruption Syndrome in 53 Postpubertal Patients: Factors Influencing the Heterogeneity of Its Presentation

**DOI:** 10.1371/journal.pone.0053189

**Published:** 2013-01-07

**Authors:** Luu-Ly Pham, Pierre Lemaire, Annie Harroche, Jean-Claude Souberbielle, Raja Brauner

**Affiliations:** 1 Université Paris Descartes, Sorbonne Paris Cité, and Fondation Ophtalmologique Adolphe de Rothschild, Paris, France; 2 Grenoble-INP/UJF-Grenoble 1/CNRS, G-SCOP UMR5272, Grenoble, France; 3 AP-HP, Hôpital Necker-Enfants Malades, Service d’Explorations Fonctionnelles, Paris, France; The Chinese University of Hong Kong, Hong Kong

## Abstract

**Background:**

Pituitary stalk interruption syndrome (PSIS) may induce an isolated growth hormone (GH) deficiency or multiple hypothalamic-pituitary (HP) deficiencies. Patients with multiple HP deficiencies, primarily those with adrenocorticotropin (ACTH) deficiency, are at increased risk of morbidity and mortality. Our objective was to identify the factors influencing each symptom and the MRI features of the syndrome to enhance its diagnosis and genetic analysis.

**Methods:**

This study was a retrospective, single-center, case-cohort study of 53 patients with PSIS who had reached pubertal age.

**Results:**

Patients were classified as having an isolated GH deficiency (n = 24, Group 1) or HP deficiencies (n = 29, Group 2); of these, 19 had complete HP deficiency, and 10 had GH deficiency associated with TSH (n = 4), TSH and ACTH (n = 3), TSH and gonadotropin (n = 1) deficiencies or amenorrhea (n = 2). The following features were less frequent in Group 1 than in Group 2: breech presentation (4% vs 35%, P = 0.008), hypoglycemia (0% vs 59%, P<0.00001), micropenis (13% vs 69%, P<0.003), hypothalamic origin (0% vs 52%, P<0.000001), ophthalmic malformation (8% vs 38%, P<0.02) and psychomotor delay (0% vs 31%, P<0.004). The frequencies of all other malformations were similar in both groups (37% vs 59%). A visible pituitary stalk was characteristic of patients belonging to Group 1 (P<0.0002). The GH peak was greater in Group 1 than in Group 2 (P<0.0003), as was the anterior pituitary height (P = 0.01).

**Conclusion:**

The factors that best discriminate patients with multiple HP deficiencies from those with an isolated GH deficiency are breech presentation, hypoglycemia, and micropenis. No patient with an isolated GH deficiency had psychomotor delay, but associated malformations and/or syndromes, with the exception of ophthalmic disorders, occurred with similar frequencies in both groups. We have also shown that each of the above characteristics is associated with a given HP deficiency and/or malformation/syndrome in the majority of cases.

## Introduction

Growth hormone (GH) deficiency is diagnosed by a low GH peak after two pharmacological stimulation tests. This diagnosis must be followed by additional testing: magnetic resonance imaging (MRI) to look for a hypothalamic-pituitary (HP) lesion (tumor or cyst) or pituitary stalk interruption syndrome (PSIS) and an evaluation of the other HP functions to distinguish an isolated GH deficiency from multiple HP deficiencies. Indeed, patients with a multiple HP deficiency, particularly those with an adrenocorticotropin (ACTH) deficiency, are at increased risk of morbidity and mortality, primarily from hypoglycemia and adrenal insufficiency [1], [2].

PSIS is diagnosed on the basis of the classic triad: interrupted pituitary stalk, absent or ectopic posterior pituitary and anterior pituitary hypoplasia or aplasia [3], [4], [5]. However, PSIS is heterogeneous in its MRI appearance [5] and clinical-biological presentation. Thus, there are variations in the height of the anterior pituitary (from absence to normal), the appearance of the posterior pituitary lobe (ectopic at the base of the hypothalamus or along the pituitary stalk, absent or normal) and the form of the pituitary stalk (interrupted, thin, absent, large or normal). The abnormality can be limited to an ectopic posterior pituitary.

The clinical and biological presentations of patients with PSIS also varies, with differences according to the birth data, the precocity and type of presenting symptom, the isolated or multiple nature of the HP deficiency, the hypothalamic or pituitary origin of the deficiency, and the associated malformations, particularly ophthalmic, and/or syndromes. We [6] have shown that patients with multiple HP deficiencies are more likely to have associated malformations than those with an isolated GH deficiency. Similarly, Reynaud et al [7] showed that multiple HP deficiencies were significantly more common in patients with extra-pituitary malformations (87.5%) than in those without (69.5%). By contrast, Simon et al [8] found no difference in the endocrine profiles of patients with and without birth defects.

This heterogeneity of clinical and biological presentations and MRI features of patients with PSIS is most likely due, in part, to the multiple mechanisms of PSIS. In addition, among patients with PSIS, those suffering from an isolated GH deficiency may develop delayed HP deficiencies or undergo abnormal puberty. Because patients with congenital HP deficiency [9], [10] and many of those with PSIS [11] are more likely to have been delivered by breech or Cesarean section and/or have suffered from neonatal hypoxemia than the general population, PSIS has been considered as secondary to these events. However, the association of this syndrome with micropenis and/or cryptorchidism as well as the occurrence of familial or syndromic forms suggest an antenatal origin, in which the abnormal birth data are a consequence and not a cause of PSIS. The GH-N, GH releasing hormone (GHRH) receptor, and Pit-1 genes do not seem to be implicated in PSIS [6]. Abnormal genes (HESX1 [7], [12], *LHX4* [13], *SOX3* [14], and recently *PROKR2* [15]) have been reported in rare cases of PSIS.

We analyzed data from 53 patients with PSIS who had reached pubertal age. Our objective was to identify the factors influencing each clinical and biological symptom and MRI feature. We focused on the roles of the HP deficiencies and the associated malformations or syndromes. We used our findings to identify symptoms indicative of the type of HP deficiency. This classification system is important for the early diagnosis of the deficiencies, primarily ACTH, in neonates and infants and for the prevention of life-threatening episodes.

## Materials and Methods

### Ethics Statement

Written informed consent for the evaluations was obtained from the children’s parents and included in the children’s hospital medical records. All clinical investigations were conducted according to the principles expressed in the Helsinki Declaration. The Ethical Review Committee (Comité de Protection des Personnes, Ile de France III) stated that “this research was found to conform to generally accepted scientific principles and research ethical standards and to be in conformity with the laws and regulations of the country in which the research experiment was performed”.

### Patients

This retrospective, single-center, case-cohort study was performed using 53 patients (29 boys) from a total of 85 patients monitored for HP deficiency with PSIS by a senior pediatric endocrinologist (R Brauner) in a university pediatric hospital from 1982 to 2011. Only those patients who had reached pubertal age (over 11 years for girls and 13 years for boys) with conclusive clinical features of puberty were included; 32 were thus excluded. Some patients had been included in previous clinical-biological studies analyzing the associated malformations: 26 observed before 1996 [6], 2 in Fanconi’s anemia studies [16], [17], one in the unic reported case of Diamond-Blackfan anemia and PSIS [18].

### Methods

The history of each patient, including consanguinity and familial forms of diseases, was recorded. Pre- and perinatal histories were reviewed. We recorded gestational age (<37 or >41 weeks), delivery by breech and/or Caesarean section, the indications for the Caesarean section, perinatal abnormalities (Apgar score <6 at 5 min and/or neonatal resuscitation), height and weight at birth, micropenis in boys, hypoglycemia and the nature of the presenting symptom. Intrauterine growth retardation (IUGR) was defined as weight or height at birth below the 3^rd^ percentile for gestational age [19]. Micropenis was defined as a penis length of less than 30 mm. The presence or antecedence of cryptorchidism was recorded. Hypoglycemia was defined as a blood glucose concentration below 3 mmol/L after 2 days of age.

Pituitary height was evaluated as previously reported [20]. Decreased growth rate was defined as a height velocity during the previous year of more than one standard deviation score (SDS) below the mean for chronological age or a decrease in height SD of more than 0.5 over one year [21]. The criterion for diagnosing GH deficiency was a GH peak response of less than 6.7 ng/mL after two pharmacological stimulation tests, excluding the response to GHRH, or during spontaneous hypoglycemia. A GHRH test was performed in 22 patients. Pituitary functions other than GH were evaluated by basal blood cortisol at 08.00 h, free thyroxin (FT4), prolactin, and the thyroid-stimulating hormone (TSH) response to thyrotropin-releasing hormone (n = 34). The plasma and urinary osmolalities after water deprivation for 12 h were normal in the first 29 patients evaluated, showing a normal concentration capacity. Plasma and urinary osmolalities were therefore not evaluated in the remaining patients. The normal limits were 12 to 28 pmol/L for plasma FT4, 0.6 to 4 mU/L for basal plasma TSH, 14±7 mU/L for peak TSH, and <10 mU/L for TSH at 120 minutes after the thyrotropin-releasing hormone test. Limits also included 5–25 µg/L for basal prolactin, except for neonates, who had higher concentrations. ACTH deficiency was diagnosed by basal plasma cortisol values below 40 µg/L in neonates and below 80 µg/L in older children, by no increase during hypoglycemia, and by low ACTH.

Advanced puberty is defined as the onset of puberty between 8 and 10 years of age in girls and between 9 and 10 years of age in boys. Puberty was considered to be abnormal when pubertal development was absent in patients of pubertal chronological and bone ages or had not progressed, including the development of primary or secondary amenorrhea. Gonadotropin deficiency was diagnosed by an absent or partial gonadotropin (luteinizing hormone, LH, and follicle-stimulating hormone, FSH) response to a gonadotropin-releasing hormone (GnRH) stimulation test.

Complete HP deficiency was diagnosed by deficiencies in GH, TSH, ACTH and LH/FSH.

The following features were considered to suggest a hypothalamic, rather than a pituitary, origin of the HP deficiency: an increased and/or prolonged TSH response to the thyrotropin-releasing hormone test and/or increased plasma basal prolactin concentrations, except in neonates. The GH response to the GHRH test was not used to discriminate between hypothalamic and pituitary origins because it depends on the age at evaluation and on the capacity of the pituitary to increase GH production [22]. The follow-up for each patient included measurements of plasma FT4 and cortisol concentrations every one to two years, if their concentrations had previously been normal, to diagnose delayed deficiencies.

Plasma hormone concentrations were measured using different immunoassays during the study period. Each new assay method for a given hormone was always cross-correlated with the previous method to ensure that the results were comparable throughout the whole period.

### Statistical Analysis

The results are expressed as the mean±SD and as percentages. For each variable, a Mann-Whitney U-test was performed, so as to detect differences of distributions from one group to the other. In the case of malformations, the test was carried out based on whether there were a malformation or not.

## Results

The patients were classified according to their status at the last evaluation. Group 1 had an isolated GH deficiency (n = 24, 45%, 15 boys, Table 1), and Group 2 had multiple HP deficiencies (n = 29, 55%, 14 boys, Table 2). These 29 patients included 19 with a complete HP deficiency and 10 with a GH deficiency associated with other deficiencies, including TSH (n = 4), TSH and ACTH (n = 3), TSH and gonadotropin deficiencies (n = 1, associated with primary testicular deficiency) and amenorrhea (n = 2).

**Table 1:** Characteristics of the patients with isolated GH deficiency.

Case	Age at diagnosis	Micropenis	Malformation	GH peak	Anterior pituitary
	yr		Syndrome	ng/mL	mm
1	1		DiGeorge syndrome	2.9	1.2
2	1.3	0		2.9	2.7
3	1.7		Cheek hamartoma	1.3	3
4	2.6	1	Diamond-Blackfan anemia, microphtamia	1.5	2
5	3.1	0		4,4	3.5
6	3.1			1.8	2
7	3.2	0		3.5	4
8	3.6	0	Palate and pharyngeal malformation	1.8	2
9	3.7	1		1.8	4
10	3.7	0	Temporal arachnoid cyst, dural ectasia	2	3
11	3.7			2.5	4
12	4.1			1.7	5
13	4.5			3.3	4
14	5	0	Arnold Chiari syndrome	3.2	3.5
15	5.5	0		1.3	3
16	6.4	0		2	4
17	6.8	0	Anorectal malformation, perineal angioma, scrotal bifidity	3	NA
18	7.1	0		1.3	4
19	7.8			3.8	5
20	8.1	0		2.4	2.5
21	8.7			1	4
22	9.4		Fanconi’s anemia, microphtalmia	1.4	1
23	13.5	0	Arnold Chiari syndrome	4.9	2
24	16.6	0		2.8	1.5

Breech delivery, low Apgar score and IUGR only in case 8.

Cesarean section in case 15 because his mother’s haemophilia.

Cryptorchidism in cases 8 and 17.

**Table 2:** Characteristics of the patients with multiple HP deficiencies.

Case	Age atdiagnosis, yr		Breechpresentation			Low Apgarscore			Hypoglycemia	Micropenis	Cryptorchidism	Hypothalamicorigin	MalformationSyndrome	Ophthalmicmalformation	Psychomotordelay	Anterior pituitary mm	GH peakng/mL	LH peakU/L	FSH peakU/L
**Complete HP deficiency**																			
25	0		0		0				1			0	Sella turcica absent, clitoral aplasia		0	0	0.3	<0.2	0.42
26	0		0		1				1	1	0	1			0	0	3.8	0.8	0.7
27	0.2		1		1				1	0	1	0	Arnold Chiari syndrome	Ptosis	0	0.5	5.3	5.4	4.3
28	2		1		0				1			1			0	3	0	0.37	<0.5
29	2.1		0		1				1			1		Unilateral nerve atrophy	0	2	2.6	1.6	0.6
30	2.2		0		0				1	1	0	0			0	2	0.4	0.2	0.2
31	2.5		0		0				1	1	1	1	Diabetes mellitus		0	2	1.3	8.4	3.7
32	2.7		1		0				1	1	0	1	Cerebral		0	2	0.7	0.85	2.2
33	3.7		0		1				1			1			1	0	0	0.39	<0.2
34	3.8		0		0				1	1	0	1	Cerebral		1	5	1	<0.2	<0.2
35	4.2		0		0				1			0		Bilateral nerve atrophy	1	2	1	17	12
36	5.1		1		0				1	0	1	1			0	NA	0.3	<0.2	<0.2
37	5.7		1		0				0			1		Unilateral nerve atrophy	0	4	0	<0.4	<0.4
38	8.3		0		1				0	1	1	0	Cerebral	Ptosis	1	0	0.7	<0.2	1.5
39	8.9		1		1				0			1			0	1	0.7	0.2	<0.4
40	9.2		1		0				NA	NA	NA	0			0	NA	0	<0.4	<0.4
41	13.3		1		0				0			1	Diabetes mellitus, Cerebral		1	1	0.4	0.3	0.4
42	18.5		0		0				0			0		Bilateral nerve atrophy	0	4	0	21	11
43	27		0		0				NA			0			0	NA	0.2	<1	<1
**GH and TSH deficiencies**																			
44		2.3		1			0		0	0	0	1			0	3.5	3		
45		4		0			0		0			0	Transient cardiomyopathy	Strabismus	0	0	1.2	37	21
46		6.2		1			0		1			0	Cerebral	Strabismus	1	2	3.4	12	12
47		10.6		0			0		0	1	1	1	Fanconi’s anemia	Microphtalmia	1	4	0.6		
**GH,TSH and ACTH deficiencies**																			
48		0.9		0			0		1	1	1	1	Olfactory bulbs aplasia	Bilateral nerve atrophy	1	2	2	14.5	2.4
49		10.5		0			0		1	0	0	0			0	1	2.7		
50		13.5		0			0		0			0			0	4	0.17	12.5	10.5
**GH,TSH and gonadotropins deficiencies**																			
51		4		0			0		1	1	1	1	Cerebral	Microphtalmia	1	3	0	0.46	<0.2
**GH and amenorrhea**																			
52		0.8		0				0	1			0			0	0.5	2.8	4.8	6.4
53		11.2		0				0	0			0			0	2.8	0.5	0.9	3.7

Prematurity in cases 27,32,41.

Cesarean section in cases 25,29,48,49.

IUGR in case 40.

Amenorrhea primary in cases 29,35,42,43 and 52 and secondary in case 53.

### Isolated GH Deficiency (Table 1)

There was consanguinity in one cases (case 21), but none of the isolated GH deficiency cases were familial. No peculiar perinatal features were observed, with the exception of one boy (case 8) born by breech delivery who had a low Apgar score, IUGR (unique in this group) and cryptorchidism; he had transient low FT4 (8 pmol/L) and low TSH that was treated with thyroxin. This patient and one other (case 13) had advanced puberty. The only Caesarean section was performed because of the mother’s hemophilia.

GH deficiency and PSIS were diagnosed at 5.6±3.7 years. The presenting symptom was a decreased growth rate, which began at 1.9±1.7 (range, 0.3 to 7) years in all but one child, who presented with micropenis at 3.7 years (case 9). Only one other boy, who had Diamond-Blackfan anemia (case 4), presented micropenis in this group.

There were no cases of hypoglycemia. Six of seven patients evaluated with the GHRH test had a GH peak greater than 10 ng/mL; the seventh was 24 years of age at the time of the GHRH test. In the six patients, there were no biological signs suggesting a hypothalamic origin other than the normal GH response to the GHRH test. Nine patients had an associated malformation or syndrome (Table 1). The patients with Fanconi’s or Diamond-Blackfan anemia also had microphthalmia, and the boy had micropenis. No other patient in this group had an ophthalmic malformation. Two boys had cryptorchidism, including one with pelvic malformation (case 17). No patient had psychomotor delay.

All patients underwent normal pubertal development, with regular menses in the 9 girls.

MRI studies showed that the posterior pituitary was ectopic at the base of the hypothalamus (n = 16) or along the pituitary stalk (n = 2), not visible (n = 4), or normal (n = 1, associated with a non-visible pituitary stalk). The pituitary stalk was interrupted (n = 9), not visible (n = 3), large (n = 4), thin (n = 6), or normal (n = 2, associated with an ectopic posterior pituitary). The anterior pituitary was visible in all patients.

### Multiple HP Deficiencies (Table 2)

There was no consanguinity. One girl (case 45) and her paternal aunt had PSIS (case 43). The mother of one boy (case 51) also had an adult height of 139 cm, and her plasma insulin-like growth factor 1 was very low (30 ng/mL) at the age of 30 years of age. This finding suggested a GH deficiency, but MRI was not performed.

Three patients were born prematurely, and four were born by Caesarean section.

Among the 10 born in a breech presentation, 8 had complete HP deficiencies and 2 had a TSH deficiency. Six had a low Apgar score, 4 of whom had no visible anterior pituitary tissue upon MRI.

HP deficiencies and PSIS were diagnosed at 6.7±5 years. The presenting symptom was decreased growth rate in 21 (72%) patients, which began at 1.7±1.7 (0 to 5) years, hypoglycemia in 6 (21%, including 3 neonates) patients, and micropenis in 2 (7%) patients.

More than half the patients (17; 58.6%) had symptomatic or diagnosed hypoglycemia before they were evaluated for GH secretion; all but three had an ACTH deficiency. Hypoglycemia was diagnosed before 6 months of age in 10 patients, but it led to the diagnosis of HP deficiency in only 5; the others were diagnosed later because of their decreased growth rate. Of the 14 boys, 9 had micropenis, and 7 had cryptorchidism. Fifteen patients had biological signs suggesting a hypothalamic origin (See Methods).

Seventeen patients had an associated malformation or syndrome. Eleven had an ophthalmic malformation (detailed in Table 2). Nine had a psychomotor delay, associated in five patients with an intracranial malformation and in one with Fanconi’s anemia. Two developed diabetes mellitus.

MRI studies showed that the posterior pituitary was ectopic at the base of the hypothalamus (n = 22) or along the pituitary stalk (n = 1) or was not visible (n = 3). The pituitary stalk was interrupted (n = 15), not visible (n = 13) or thin (n = 1). Five patients had no visible anterior pituitary, and among them, 4 had complete HP deficiency.

Puberty was normal in 7 patients (4 boys and 3 girls, cases 44 to 50) and abnormal in 22 (10 boys and 12 girls, Table 2). Among the boys with normal puberty, two had micropenis and cryptorchidism, which were associated in one with Fanconi’s anemia and followed in the other by advanced puberty. The 10 boys with abnormal puberty included 7 with micropenis, which was associated with cryptorchidism in 3. Two of the remaining three had isolated cryptorchidism, and this information was unavailable in the tenth boy. Among the 10 boys, 8 had LH and FSH peaks below 1 U/L after the GnRH test; 2 had LH/FSH peaks of 5.4/4.3 and 8.4/3.7 U/L, plasma testosterone concentrations that remained below 0.5 ng/mL, and micropenis (one) and/or cryptorchidism (both). The 12 girls with abnormal puberty included 6 with no pubertal development and low LH and FSH peaks and 6 whose pubertal development was incomplete (5 with primary and 1 with secondary amenorrhea). The latter 6 girls included 3 with complete LH/FSH deficiencies and 3 with LH (4.8 to 21 U/L) and FSH (6.4 to 12 U/L) peaks after the GnRH test and a plasma estradiol concentration of 12 to 23 pg/mL.

### Group Comparisons (Table 3)

The most discriminatory variables suggesting a patient belonged to Group 2 were breech presentation (P = 0.008), hypoglycemia (P<0.00001), micropenis (P<0.003), cryptorchidism (P = 0.003), and hypothalamic origin (P<0.000001). The strongest indicator that a patient had a complete HP deficiency was a presenting symptom other than a decreased growth rate (P = 0.01). The GH peak was greater in patients with an isolated GH deficiency than in those with multiple HP deficiencies (P<0.0003), as was the anterior pituitary height (P = 0.01). Five Group 2 patients had no visible anterior pituitary in MRI studies, but all Group 1 patients had a visible anterior pituitary (P<0.0002).

**Table 3:** Comparison between isolated GH deficiency (Group 1) and multiple HP deficiencies (Group 2).

	All PSIS	Group 1	Group 2	P
	n = 53 (100%)	n = 24 (45%)	n = 29 (55%)	
Boys	29 (54.7)	15 (62.5)	14 (48.3)	NS
Age at diagnosis, yr		5.6±3.7	6.7±5	NS
Breech presentation	11 (20.7)	1 (4.1)	10 (34.5)	0.008
Caesarean section	5 (9.4)	1 (4.1)	4 (13.8)	NS
Low Apgar score	7 (13.2)	1 (4.1)	6 (20.7)	NS
Hypoglycemia	17 (32)	0 (0)	17 (58.6)	<0.00001
Micropenis (% boys)	11 (39.3)	2 (13.3)	9 (69.2)	<0.003
Cryptorchidism (% boys)	9 (24.3)	3 (20)	7 (53.9)	0.003
Hypothalamic origin	15 (28.3)	0 (0)	15 (51.7)	<0.000001
GH peak, ng/mL		2.4±1.1	1.2±1.4	<0.0003
Anterior pituitary height, mm		3.1±1.1	2±1.5	0.01
Malformations/syndromes	26 (49)	9 (37.5)	17 (58.6)	NS
Ophthalmic malformations	13 (24.5)	2 (8.3)	11 (38)	<0.02
Psychomotor delay	9 (17.0)	0 (0)	9 (31.0)	<0.004

The malformations or syndromes occurred with similar frequencies in both groups, with the exceptions of psychomotor delay, which occurred only in Group 2 (P<0.004), and ophthalmic malformations, which were more frequent in Group 2 (P<0.02).

## Discussion

Among patients with PSIS, we have shown that the factors that best discriminate patients with multiple HP deficiencies from those with an isolated GH deficiency are breech presentation, hypoglycemia, and micropenis, while a visible pituitary stalk suggests an isolated GH deficiency. No patient with an isolated GH deficiency had psychomotor delay. Associated malformations and/or syndromes occurred with similar frequencies in both groups, with the exception of ophthalmic disorders, which were more frequent in Group 2. We have also shown that each of breech presentation, hypoglycemia, and micropenis is associated with a given HP deficiency and/or malformation/syndrome in the majority of cases.

### Birth Data

The frequencies of IUGR and Caesarean deliveries were similar in the isolated and multiple HP deficiencies, but breech deliveries were significantly more frequent in patients with multiple deficiencies. Thus, 35% of these patients were delivered in a breech presentation compared to only 4% of those with an isolated GH deficiency, a frequency similar to that of the general population in France, [23] while the proportion of Caesarean deliveries was not greater [24]. While most reports indicate that patients with multiple deficiencies are more likely have a breech presentation [8], [9], [25], [26], Melo et al [27] found no differences in the frequencies of cephalic and breech deliveries in patients with an isolated GH deficiency and in those with multiple HP deficiencies in PSIS. The causes of a breech presentation are unknown. Rayl et al [28] performed a population-based, case-control study of risk factors for breech presentation. They found that a low birth weight, prematurity, primiparity, and older maternal age were associated with an increased risk. After controlling for these factors, they found that hydrocephalus (OR 11.4), any recorded congenital malformation of the infant and maternal diabetes were also associated with breech presentation. We found no association between breech presentation and malformations. All 11 patients who presented as breech in our study had low plasma FT4 concentrations, including the one patient with an isolated GH deficiency and a transient low FT4 concentration at diagnosis. Two others had GH and TSH deficiencies, while the remaining 8 had complete HP deficiency. However, the high frequency of this presentation in patients with congenital peripheral hypothyroidism has not previously been reported, and the presentation has not been described in reports of patients with an isolated TSH deficiency [29]. Six of the 7 patients who had a low Apgar score had complete HP pituitary deficiencies; 4 out of these 6 had neonatal hypoglycemia. All had very small or no visible anterior pituitary tissue in MRI studies. Albertsson-Wikland et al [30] found that Apgar scores below 7 at 5 min were more frequent in patients who later developed an idiopathic GH deficiency (5.2%) than in the total Swedish newborn population (1.2%). Murray et al [31] examined 67 patients with an ectopic posterior pituitary, 30 of them aged less than 14 years. They found that patients with an isolated GH deficiency were less frequently admitted to neonatal intensive care than those with multiple HP deficiencies, while their age at first referral and the size of their anterior pituitary were significantly greater than for those with multiple deficiencies.

### Precocity and Type of the First Symptom

The most common presenting symptom in patients with an isolated GH deficiency was decreased growth rate, with the exception of one patient, who presented with micropenis. For patients with multiple HP deficiencies, the presenting symptom was a decreased growth rate in 72%, hypoglycemia in 21%, and micropenis in 7%. While 59% of these Group 2 patients suffered from hypoglycemia, this symptom did not initiate the HP evaluation in several cases despite its association with micropenis in the majority of the boys. The diagnosis was made later as a result of a decreased growth rate. All but three patients with hypoglycemia had an ACTH deficiency. Hypoglycemia suggests a cortisol deficiency [32] and may be associated in neonates with prolonged jaundice (three of our patients) and/or low blood pressure. Profound persistent hypoglycemia is a clear warning when unexplained by other neonatal conditions. Because hypoglycemia is the most important stimulus for GH and ACTH secretion, these hormone plasma concentrations should be measured during spontaneous hypoglycemia, whatever the age of the patient. The association of hypoglycemia and micropenis in neonates and infants is highly suggestive of an HP deficiency. Its association with breech presentation and/or malformations, mainly ophthalmic, is also suggestive. Taback et al [1] reported a surprisingly high proportion of deaths (9/37) caused by an adrenal crisis and hypoglycemia in children administered GH for a GH deficiency; these included 7 with a congenital HP deficiency and 2 with craniopharyngioma. Mills et al [2] analyzed the mortality of children treated with GH, showing that those with hypoglycemia had a ninefold greater risk, primarily in the first few years of life and of treatment, and that many of these deaths resulted from a combination of hypoglycemia, seizures, and possible adrenal insufficiency in children with hypopituitarism.

### Associated Malformations

More than one third (37%) of the patients with an isolated GH deficiency and 59% of those with multiple HP pituitary deficiencies had malformations and/or congenital diseases. Simon et al [8] reported that 42% and 58% of their patients with isolated and multiple HP deficiencies, respectively, also had extrapituitary birth defects. Psychomotor delay, a symptom frequently associated with cerebral malformations, occurred in 31% of cases. In these patients with multiple HP deficiencies, hypoglycemia and/or adrenal deficiency may have contributed to the psychomotor delay.

### Puberty

All the boys with delayed puberty had micropenis and/or cryptorchidism. The other 5 boys with micropenis and/or cryptorchidism had Diamond-Blackfan anemia, Fanconi’s anemia, or unilateral cryptorchidism with a pelvic malformation or developed advanced puberty (two cases). Rottembourg et al [33] reported that 9 of 11 evaluated boys with gonadotropin deficiency in PSIS had micropenis and 5 had cryptorchidism, which was isolated in 1, while only 1 of 4 boys with spontaneous puberty had micropenis and cryptorchidism. Only 1 (with an isolated GH deficiency) of the 12 girls they studied underwent normal puberty and had regular menses; 7 had no pubertal development and low LH (below 0.8 U/L) and FSH (below 1.5 U/L) peaks, while the remaining 4 had incomplete pubertal development and primary or secondary amenorrhea. In these, the median peaks were 30 U/L for LH and 11 U/L for FSH. We found similar features in girls with multiple HP deficiencies, while the 9 girls with an isolated GH deficiency had normal puberty and regular menses.

### Conclusion

Every newborn should be examined for the following characteristics suggesting multiple HP deficiencies due to PSIS: breech presentation, low Apgar scores or neonatal resuscitation, hypoglycemia, prolonged jaundice, and micropenis that is isolated or associated with cryptorchidism. The presence of ophthalmic malformations should also be determined. Each characteristic is associated with a peculiar hormonal deficiency or is a part of syndrome. A breech presentation suggests a TSH deficiency, while micropenis and/or cryptorchidism suggest gonadotropin deficiency. Hypoglycemia is primarily associated with an ACTH deficiency. The early diagnosis of ACTH deficiency is essential to the initiation of Hydrocortisone® treatment in emergency settings. Parents and patients should be carefully and repeatedly informed of the need to increase the Hydrocortisone® dose in response to specific events, such as stress, and to replace it with injections if there are any gastrointestinal symptoms or the need for anesthesia arises. These measures may reduce the delay in diagnosis and decrease the morbidity and mortality in this population.

This study is limited by the fact that all the patients were observed by a single physician. However, this bias may be minimized by the recruitment procedures, which included all patients evaluated for PSIS by the physician. We report our findings to increase physician awareness of this disease, particularly that of neonatologists and those working in emergency departments.
